# Entity-aware pathology workflow informatics: Structured evidence mapping and a reference architecture for governed orchestration

**DOI:** 10.1016/j.jpi.2026.100720

**Published:** 2026-09-14

**Authors:** Ao Li, Peter O'Loughlin, Lin Yue, Wei Emma Zhang, Bernadette Swart, Weitong Chen

**Affiliations:** a Australian Institute for Machine Learning, Adelaide University, Adelaide, SA 5005, Australia; b SA Pathology, Frome Road, Adelaide, SA 5000, Australia

**Keywords:** Pathology informatics, Workflow orchestration, Lab information systems, Object-centric process mining, Digital twin, Clinical governance

## Abstract

**Objective:**

To map evidence relevant to representing and governing pathology workflows spanning cases, specimens, blocks, slides, scans, and reports, and to derive a reference architecture.

**Methods:**

We performed structured evidence mapping with purposive horizon scanning. Searches of Scopus, PubMed, and IEEE Xplore yielded 74 primary database records; Google Scholar and citation tracking contributed 24 supplementary records. Each record contributed one tuple across data acquisition, control-flow reconfiguration, execution autonomy, and validation environment (D1–D4), an author-developed operational taxonomy. Unretained historical search details are reported as a reproducibility constraint, not reconstructed as original methods.

**Results:**

The 98-record evidence base spans 1993–2026. Within the 74-record primary corpus, 47 records had dashboard-level execution; validation was split between real-world deployment (26) and conceptual evidence (25). Under study-defined filters, 8/74 records met all 4 minimum capability conditions, 2/74 also showed closed-loop physical actuation, and 1/74 combined full autonomy with real-world deployment. These study-defined filters derive from the original single-reviewer D1–D4 coding. An independent blinded 50-record verification yielded 81.6% inclusion agreement (κ = 0.627) and 80.0% exact D1–D4 field agreement, but did not constitute full-corpus independent reproduction. Supplementary records informed emerging automation requirements. The mapped gaps informed an architecture comprising semantic perception, entity-aware process state, bounded orchestration, pre-action command governance, governed actuation, and conditional local execution support; a safety-and-constraints sandbox is one candidate realization of the governance function.

**Conclusion:**

The architecture is an evidence-derived design target, not a deployed or validated system. Evaluation should test event provenance, case-to-slide relationships, robustness to missing or shifted inputs, command constraints, human override, and staged operational performance before autonomy claims.

## Highlights

• A 98-record map separates 74 primary from 24 horizon-scan records.
• Each record contributes one author-developed D1-D4 coding tuple.
• Eight of 74 primary records met all four study-defined capability filters.
• The architecture keeps orchestration technologies implementation-neutral.
• Deployment requires failure tests, oversight, and prospective validation.

## Graphical abstract

**Figure:**
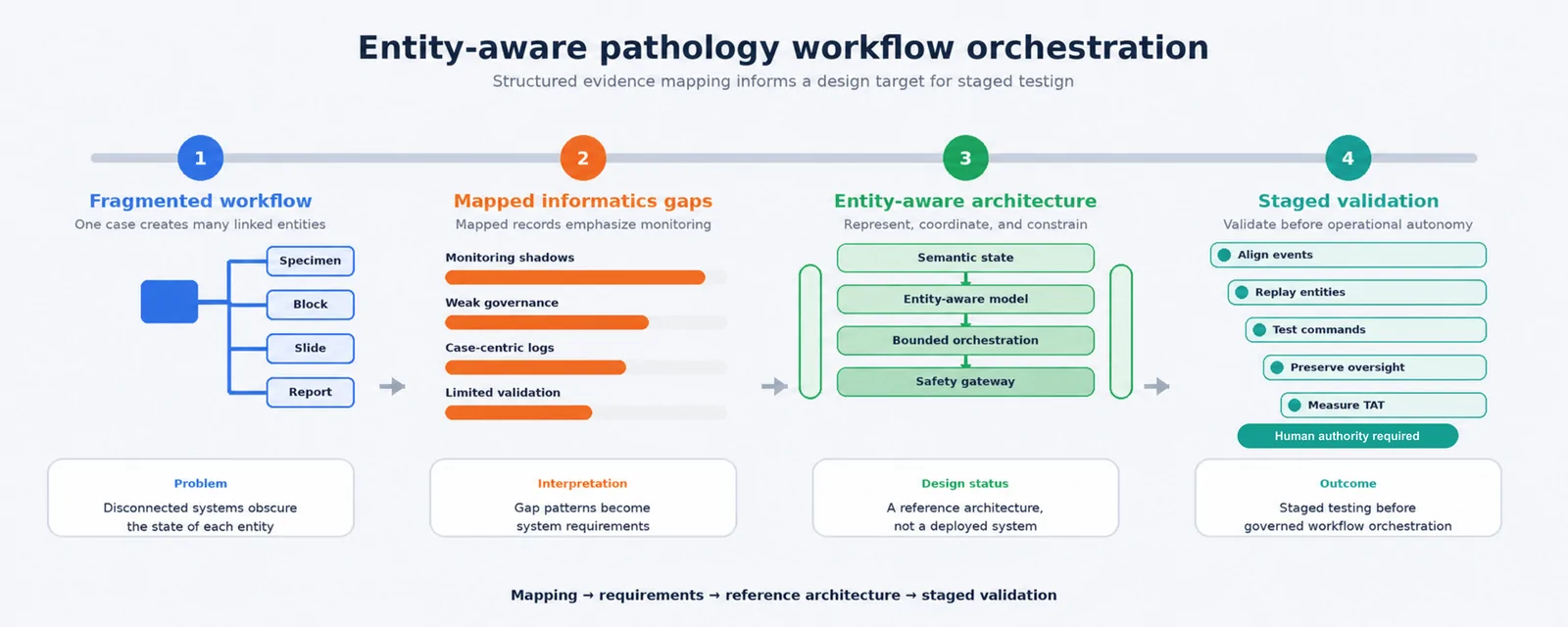

## Introduction

### Pathology workflow informatics as the primary problem domain

Core labs process standardized, high-volume fluid pipelines under continuous turnaround-time (TAT) pressure, relying on total lab automation (TLA), middleware, and lab information system (LIS)-connected analyzers.^1^, ^2^ However, these workflows still require active monitoring and process improvement when specimen priorities, workload peaks, analyzer availability, or local routing rules disrupt expected TAT. Middleware-LIS and digital-shadow studies indicate that operational monitoring supports bottleneck identification and optimization, but does not establish autonomous rerouting around physical workflow failures.^3^, ^4^

Anatomical pathology exposes a more severe representation problem. A single diagnostic case divides into multiple specimens, blocks, slides, stains, scans, pathologist assignments, reports, and amendments. This one-to-many hierarchical fission challenges lab software designed around linear case records.^5^ Conventional case-centric process mining and scheduling generate spurious cycles by failing to distinguish multiple simultaneous token streams derived from one case root.^6^ This aligns with the broader object-centric process mining (OCPM) problem, where events involve several interacting objects rather than a single case identifier.^7^ Recent pathology-informatics reviews likewise identify vendor-neutral interoperability, infrastructure, implementation governance, and workflow integration as persistent barriers.^8^, ^9^, ^10^ Together, these findings motivate entity-aware workflow informatics as a pathology problem in its own right; possible transfer to other multi-entity biomedical workflows is secondary.

### Research objectives and contributions

This study reports structured evidence mapping with a targeted horizon-scanning supplement. Its objectives are to map pathology and lab-workflow evidence across four operational capability dimensions, distinguish direct pathology evidence from adjacent design precedent, and derive a traceable reference architecture for governed pathology orchestration. “Structured evidence mapping” is used deliberately: the selection and coding workflow was documented, but parts of the historical search record needed for a fully reproducible systematic search were not retained.

The framework treats preservation of multi-entity relationships as a state requirement when those relationships materially affect the decision under study. Object-centric petri nets (OCPNs), colored petri nets (CPNs), artifact-centric models, and business process model and notation (BPMN) remain candidate implementations for replay or workflow execution.^6^, ^11^, ^12^, ^13^, ^14^ Multi-agent aystems (MAS) are one candidate coordination pattern, not a mandatory architecture, and deterministic rules, centralized optimization, and workflow engines remain appropriate where scope and state are stable. Pre-action command governance is the invariant requirement; a safety-and-constraints sandbox is one candidate realization informed by prior safety-twin and clinical-governance concepts.^15^, ^16^ The contribution is the pathology-specific evidence-to-requirement derivation chain and the explicit boundary between representation requirements, candidate implementations, and validation tests; it is not a claim that the resulting architecture has been implemented or validated.

## Related work and conceptual background

### Digital shadows, digital twins, and lab workflow automation

Medical labs record operational traces across LISs, middleware, analyzers, scanners, repositories, and reporting systems. These traces enable digital-shadow systems to monitor queues, instrument status, and TAT. In core labs, such systems support bottleneck detection and workflow improvement but remain observational: they summarize or replay operations without representing derivative entities, testing alternative routing, or issuing governed commands to physical resources.^3^, ^4^

Digital-twin research indicates that twinning exceeds simple data mirroring.^17^ We therefore use *digital shadow* for one-way observation or replay without a governed command path, and reserve *operational workflow twin* for a synchronized state representation that can evaluate bounded interventions and connect approved actions to digital or physical execution. Bidirectional data exchange alone is insufficient if entity relationships, intervention logic, and command authority are not represented.

### Process mining, object-centric event logs, and entity-aware workflow representation

Process mining links event logs to observed behavior. Work on OCPM shows why healthcare processes can exceed patient- or case-centric assumptions: clinical data contain multiple object types, abstraction levels, and relationships that must be transformed into object-centric logs before OCPM applies.^18^ In pathology, a diagnostic case splits into specimens, blocks, slides, stains, scans, and reports. A single case identifier is insufficient for analyses in which derivative entities move through physical and digital subsystems at different speeds.

OCPM offers the broader methodological grounding for this problem. Traditional process-mining event logs assume that each event belongs to one case, whereas object-centric event data allow events to refer to multiple objects of different types.^7^ The object-centric event log (OCEL) provides a standard exchange format for such logs, and OCEL 2.0 extends this representation with object changes, object relationships, and relationship qualifiers.^11^, ^19^ This literature indicates that entity-aware event representation is needed before process discovery, prediction, or intervention when cross-entity dependencies can change the inferred state or permissible action.

Within that methodological family, OCPNs and artifact-centric workflow models provide formal ways to represent interacting object lifecycles.^12^, ^13^ In pathology-specific work, Stünkel et al. showed that conventional ProM algorithms can produce spurious cycles when anatomical pathology logs cannot distinguish request forms, specimens, blocks, and slides.^6^ CPNs are therefore one simulation-oriented candidate for pathology entity fission, not the uniquely required solution. The contribution of this review is to connect an implementation-neutral entity-aware state requirement to pathology synchronization, bounded coordination, command governance, and staged validation criteria.

### From monitoring to governed orchestration

Moving from retrospective monitoring to closed-loop orchestration also requires explicit control and governance assumptions. Lab automation, robotics, self-driving-lab systems, and clinical-AI governance work provide design precedents for routing, simulation, command checking, and human oversight. These adjacent-domain sources inform the requirements for workflow changes before execution in a clinical lab.

Accordingly, the literature motivates three linked requirements. First, the process representation must preserve entity identity and dependency even if the implementation uses deterministic rules, BPMN, centralized optimization, MAS, or a hybrid. Second, proposed commands require a governance layer that can reject, simulate, approve, or escalate actions before they affect specimens, instruments, or reports.^15^, ^16^ Third, local deployment support may be needed where privacy, latency, scanner/LIS locality, or trace-buffering constraints make cloud-only orchestration unsuitable.^20^, ^21^ These themes establish the conceptual basis for examining whether the mapped literature supports a progression from monitoring to governed orchestration without presupposing one control paradigm.

### Architecture alternatives and contribution boundary

Conventional workflow systems, digital shadows, process models, operational digital twins, centralized optimization, and MAS solve different problems rather than forming a single capability sequence. Table 1 states their bounded roles and the selection logic used in the reference architecture. The design is deliberately hybrid: deterministic controls remain preferred for stable safety rules; BPMN-like models make nominal workflows inspectable; optimization can rank alternatives when objectives and constraints are explicit; and MAS becomes relevant only where distributed state and local perturbations make a single decision point brittle. None of these technologies substitutes for reliable entity links or command governance.

**Table 1:** Conceptual and implementation alternatives for pathology workflow informatics.

Paradigm	Best fit	Principal limitation	Role in this architecture
Conventional rules/BPMN	Stable constraints, nominal sequence, handoffs, and human tasks	Rule growth and local exceptions; multi-entity fission may require a companion state model	Default for auditable hard constraints, fail-safe fallback, and inspectable workflow execution.^14^
Centralized scheduling/optimization	Explicit objectives, capacities, and bounded alternatives	Depends on current global state and may be brittle to missing events or rapid local disruption	Candidate planner for ranking feasible actions; outputs still require pre-action governance.^23^, ^24^
Process mining and OCPN/CPN/artifact models	Discovery, multi-object lifecycles, replay, conformance, and simulation	Models do not themselves provide clinical semantics, optimization, or device control	Candidate representations implementing the entity-aware state and replay requirements.^6^, ^12^, ^13^
Digital shadow	One-way monitoring, event reconstruction, bottleneck analysis, and replay	No governed intervention or command path	Appropriate first deployment phase and operational baseline.^3^, ^4^
Operational workflow digital twin	Synchronized state, intervention testing, and governed digital or physical action	The label is unsupported without entity links, authority boundaries, and prospective validation	Target capability definition, not evidence that the present architecture has achieved twinning.
MAS	Distributed resources, local perturbations, and asynchronous entity states	Coordination complexity, non-determinism, verification burden, and risk of emergent conflicts	Optional coordinator over a shared entity-aware state, not a default replacement for rules or optimization.^25^
Present reference architecture	Integrates pathology entity state, implementation-neutral orchestration, command governance, and staged tests	Evidence-derived synthesis without prototype, deployment, or prospective validation	Design target for comparative implementation and site-specific validation.
Gurung et al.^15^	Autonomous biofilm lab with control and safety twins	Adjacent biomedical-lab setting; no pathology workflow or clinical authority model	Pre-action safety-filter precedent; not evidence of pathology deployment.
Pańkowska et al.^14^	IoTDT–BPMN, agents, and robotic-arm model for pathomorphology	Executable single-workflow demonstration rather than corpus synthesis or multi-vendor governance	Pathology-relevant executable-model precedent and candidate implementation.
Eccher et al.^22^	Stakeholder manifesto for pathology-lab digital twins	No record-level taxonomy, executable entity contract, or evaluated command gateway	Pathology stakeholder and authority precedent; complementary to the present evidence map.

Three close precedents identified in the mapped corpus define the contribution boundary. They were selected for feature-level comparison because each overlaps at least two of the present design concerns: pathology or biomedical-lab context, executable workflow coordination, multi-entity representation, pre-action governance, or stakeholder authority. This is a bounded within-corpus comparison, not a claim that incomplete historical searches established the globally nearest prior work. Gurung et al. describe a digital-twin-based autonomous biofilm lab with a *Safety and Constraints Twin*; this is adjacent biomedical-lab evidence for pre-action filtering, not pathology deployment.^15^ Pańkowska et al. couple IoTDT-BPMN, agents, and a robotic-arm digital twin for a pathomorphological workflow, providing a pathology-relevant executable-model precedent but not a corpus-level evidence map or multi-vendor governance architecture.^14^ Eccher et al. provide a stakeholder manifesto for pathology-lab digital twins, but do not supply record-level D1–D4 coding, an object-centric event contract, or a governed command gateway.^22^ The present contribution is narrower than a validated platform: it links a structured 98-record map to pathology-specific representation, governance, and staged-test requirements. Table 1 compares both implementation paradigms and these three precedents.

## Methods

### Review design, search strategy, and data sources

We performed structured evidence mapping informed by systematic-mapping guidance^26^ and used a PRISMA 2020-adapted reporting approach for the applicable selection and reporting items. We did not prospectively register a protocol, and no separate protocol was prepared. The label *systematic review* is not used because the historical search record does not meet the reproducibility standard implied by that term. All database and supplementary searches were completed on May 1, 2026 and restricted to English-language records. Scopus, PubMed, and IEEE Xplore were searched using digital-twin and cyber-physical-system terms combined with lab medicine and pathology workflow terms. The exact Scopus expression was retained. The contemporaneous PubMed field-tag expression, IEEE Xplore interface-field settings, and original export files were not retained; Table 2 therefore reports the recorded conceptual expressions and retrieval counts rather than presenting reconstructed syntax as the original searches. Supplementary search strategies supplies clearly labelled, platform-specific rerun translations and records this limitation against the relevant PRISMA-S reporting items.^27^

**Table 2:** Recorded search strategies, conceptual query components, and initial retrieval counts (Searches completed on May 1, 2026).

Information source	Search strategy/Conceptual query structure	Records (*n*)
Scopus	**Field:** TITLE-ABS-KEY **Set A (Digital Twins & CPS):** (“digital twin*”**OR**“digital shadow*”**OR**“virtual twin*”**OR**“cyber-physical system*”) **AND** (“pathology lab*”**OR**“clinical lab*”**OR**“core lab*”**OR**“medical lab*”**OR***“laboratory medicine”*) **OR Set B (Informatics & Workflow):** (*“artificial intelligence”***OR***“machine learning”***OR***“process mining”***OR***“discrete event simulation”***OR***“ontology”***OR***“knowledge graph”*) **AND** (“pathology lab*”**OR**“clinical lab*”**OR**“core lab*”**OR**“medical lab*”**OR***“laboratory medicine”*) **AND** (“workflow*”**OR***“turnaround time”***OR***“TAT”***OR***“throughput”***OR**“bottleneck*”**OR***“scheduling”***OR***“process improvement”*)	349
PubMed	**Conceptual Expression (All Fields):** (“digital twin*”**OR**“digital shadow*”**OR**“virtual twin*”**OR**“cyber-physical system*”) **AND** (“pathology lab*”**OR**“clinical lab*”**OR**“core lab*”**OR**“medical lab*”**OR***“laboratory medicine”* OR *“lab automation”*)^a^	31
IEEE Xplore	**Conceptual Expression (All Metadata):** (“digital twin*”**OR**“digital shadow*”**OR**“virtual twin*”**OR**“cyber-physical system*”) **AND** (“pathology lab*”**OR**“clinical lab*”**OR**“core lab*”**OR**“medical lab*”**OR***“laboratory medicine”* OR *“lab automation”*)^a^	11
Google Scholar	**Targeted Horizon Scan:** (*“Agentic workflow”***OR***“Multi-agent system”***OR***“LLM agent”***OR***“Autonomous agent”*) **AND** (*“laboratory automation”***OR***“wet lab”***OR***“scientific discovery”***OR***“self-driving laboratory”*)^b^	10
Citation Tracking	Backward and forward citation tracking of seed pathology workflow literature and relevant digital twin reviews	14

a Historical platform-specific interface tags and export records were not contemporaneously retained. Full platform-specific rerun translations and syntax adaptations are detailed in Supplementary search strategies.

b Purposively retained records covering agentic workflow execution, wet-lab robotics, and autonomous lab control for requirement derivation; excluded from primary prevalence denominators.

The temporal scope is the literature corpus and derived coding assets; no institutional dataset, prospective deployment, or workflow-simulation trace was analyzed. Supplementary materials contain the extraction matrix and figure data, and the reference architecture is a design synthesis.

The primary database search identified 391 records. Supplementary searches captured emerging agentic and self-driving lab paradigms: Google Scholar retained 10 records, whereas manual and backward/forward citation searching added 14.^28^ The retained records and query concepts are documented, but the Google Scholar result order and number screened, and the citation-tracking seeds, rounds, and stopping rule, were not preserved contemporaneously. The supplementary search was therefore purposive horizon scanning rather than a reproducible comprehensive search. These 24 records were excluded from primary frequency denominators and are tracked in Supplementary Table S1 as evidence for design rationale and validation requirements. This is a study-framing constraint: the mapped corpus is a dated, structured snapshot of the retrieved literature, not proof of exhaustive field coverage.

### Eligibility criteria

Studies were eligible when they addressed at least one of four concerns relevant to closed-loop pathology automation: (1) construction or evaluation of digital twins, digital shadows, cyber-physical systems, process-mining models, or discrete-event simulations in lab environments; (2) artificial-intelligence or rule-based systems used to optimize TAT, specimen logistics, quality control, or workflow routing; (3) medical lab data standardization, semantic interoperability, ontologies, or knowledge graphs; or (4) robotic, self-driving, or agentic lab systems with architectural relevance to physical actuation.

We excluded studies whose primary contribution was image-level cancer-cell recognition without lab workflow implications, patient-level clinical twins such as tumor metabolism models, basic biomedical studies focused on chemical reactions without relevance to lab operations, short editorials without an empirical or architectural framework, and sources that did not provide enough methodological or technical detail to support coding. One reviewer screened all titles/abstracts and full texts and performed the first-pass coding. To reduce subjective drift, coding used pre-specified D1–D4 definitions, conservative category assignment, and a supplementary coding matrix that preserves study-level taxonomy assignments for transparent review. Ambiguous cases were resolved by returning to the four coding dimensions and selecting the lowest category explicitly supported by the source. The resulting frequencies describe this corpus and support requirement derivation; they are not estimates of prevalence in the broader field.

### Study selection and PRISMA 2020-adapted flow

Fig. 1 summarizes a PRISMA 2020-adapted flow for the primary database corpus and the separately tracked supplementary horizon-scanning stratum. The primary database search yielded 391 records. After removing 20 duplicates, 371 titles and abstracts were screened; 198 database records were assessed in full text, and 74 met all inclusion criteria for the primary mapping corpus. Ten Google Scholar studies and 14 manually tracked studies were retained as a separate horizon-scanning stratum, yielding a 98-record evidence base for requirement derivation. Supplementary Table S1 provides the bibliography, source route, corpus role, and D1–D4 coding for all included records. Supplementary Table S2 lists records excluded during full-text assessment and the primary reason for exclusion. The first screening stage used titles and abstracts, whereas records retained after that stage underwent full-text eligibility assessment.

**Fig. 1:**
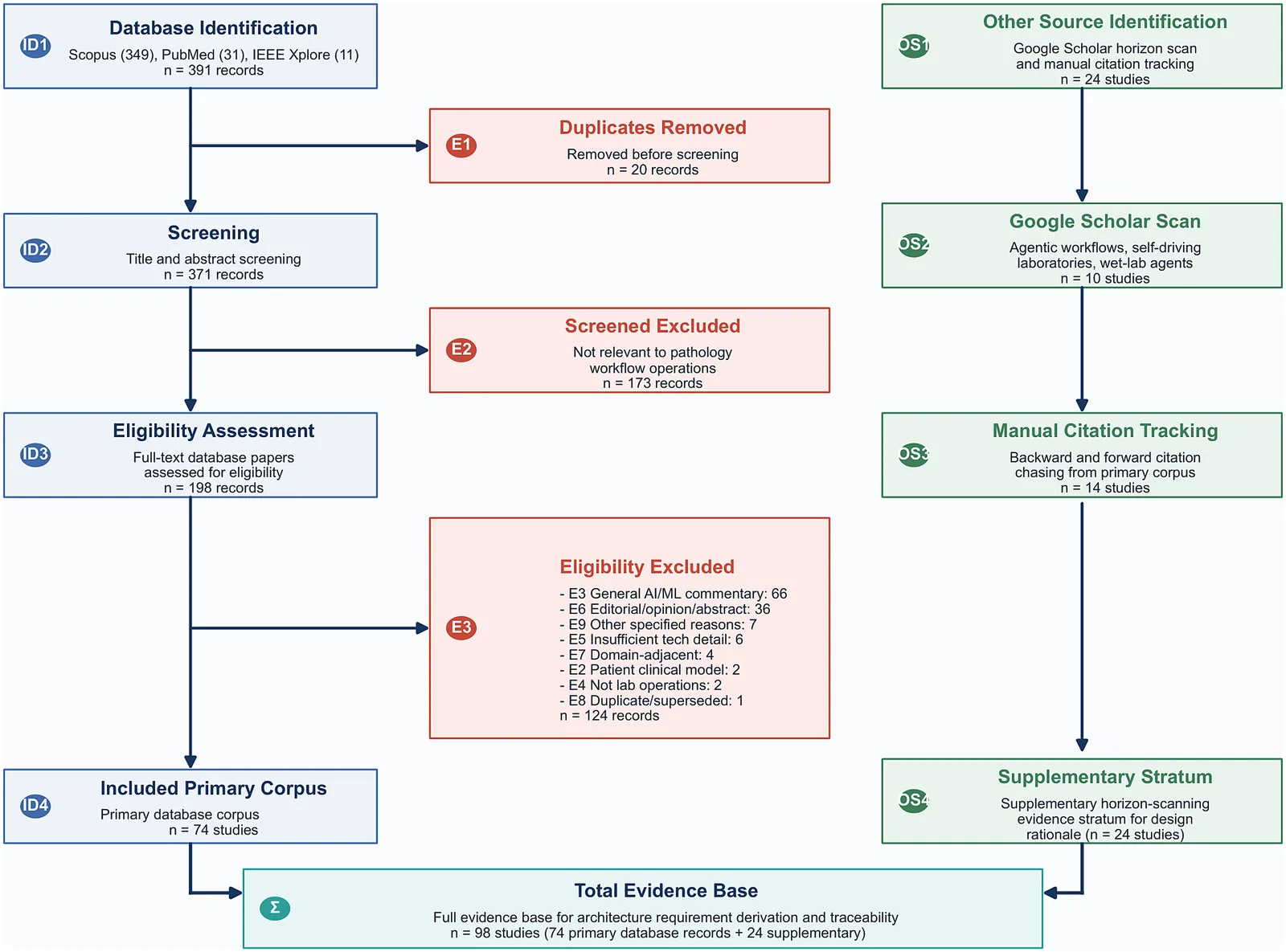
PRISMA 2020-adapted study selection flow for the primary database corpus and separately tracked supplementary horizon-scanning stratum. The primary database corpus is the denominator for primary mapping summaries; supplementary records are retained for requirement derivation and design rationale.

### Data items, collection, and synthesis

We developed the four-dimensional D1–D4 taxonomy for this study (Table 3). It is an author-developed operational classification, not an externally validated operational taxonomy. D1 records data-acquisition immediacy; D2 records the flexibility of control-flow reconfiguration; D3 records execution authority; and D4 records proximity of validation to routine operation. The dimensions were selected because a pathology workflow twin must sense lab state, decide whether and how to reconfigure workflow, execute or trigger governed physical or digital changes, and demonstrate validity beyond conceptual design.

**Table 3:** Author-developed D1–D4 operational taxonomy used for record-level coding.

Dimension	Levels	Coding interpretation
D1: Data acquisition	Offline transfer; DB polling; Event pub/sub; Real-time Internet of Things (IoT)	Static export, database polling, event subscription, or live sensor/instrument state.
D2: Control-flow	Post-mortem; Static guardrails; Predictive routing; Agentic sandbox	Retrospective logic, rule-bound control, predictive advice, or dynamically generated and safety-tested reconfiguration.
D3: Execution autonomy	Dashboard; Digital actuation; Human-in-loop; Lights-out	Human advice, digital trigger, explicit approval, or bounded operational execution.
D4: Validation environment	Conceptual; Retrospective replay; Simulation-twin; Real-world deployment	Conceptual claim, historical replay, simulation/digital-twin test, or operational evaluation.

The ordering within each dimension is operational rather than an equal-interval scale or a universal claim of technological superiority. Higher levels require stronger evidence of immediacy, reconfiguration flexibility, execution authority, or deployment proximity within that dimension. However, the categories are not assumed to be unidimensional psychometric scales: D1 combines acquisition immediacy with physical telemetry at Level 4, and D3 distinguishes digital actuation, explicit human approval, and bounded autonomous execution. Dimensions were coded independently, and no summed D1–D4 score was calculated. For every record, the reviewer assigned the highest level explicitly supported by the source; absent or ambiguous evidence was coded conservatively. The conjunctive capability filters used in the Results are transparent study-defined rules, not externally validated cut points.

Using the prespecified extraction matrix, one reviewer recorded publication year, source route, corpus role, system description, and one D1–D4 coding tuple for each included record. Study authors were not contacted; screening and extraction used no automation tools. Results were summarized using descriptive counts, cross-tabulations, and visual maps at the record level. Because the review mapped system characteristics rather than intervention effects, no effect measures, meta-analysis, formal risk-of-bias or reporting-bias assessment, certainty grading, or sensitivity analysis was undertaken.

Publication year was treated as a descriptive temporal marker. Temporal summaries therefore represent publication-year distributions of the mapped literature, not deployment timelines or longitudinal validation of the proposed architecture.

### Second-reviewer verification protocol

To evaluate screening and coding reproducibility, an independent second reviewer evaluated a stratified sample of 50 full-text records: 30 included records and 20 excluded full-text records. The assessment was performed independently and blinded to original inclusion status and author taxonomy coding.

The verification was conducted against pre-specified eligibility criteria and the standardized codebook (Supplementary Table S6). All 153 non-blank responses adhered to the prescribed controlled vocabulary. Following the blinded assessment, discordant classifications underwent structured joint adjudication against source texts to resolve borderline domain boundary and scoping ambiguities.

Inclusion and exclusion-reason agreement were evaluated using percent agreement and unweighted Cohen's kappa. For mutually included records, D1–D4 reporting used exact percent agreement and linearly weighted Cohen's kappa for ordinal Level 1–4 categories.^29^ *Insufficient Data* remained an explicit category for exact agreement but was excluded from ordinal weighted kappa calculations. Mutually included records without complete D1–D4 ratings remained missing and were not imputed. Kappa coefficients were interpreted alongside observed marginal distributions and raw agreement.^30^ Supplementary Table S7 reports the verification protocol, paired evaluation records, and dimension-specific agreement metrics.

### Architecture derivation method

The reference architecture was derived through evidence-informed requirement synthesis; deployment testing was outside scope. D1–D4 classified operational evidence for data acquisition, control-flow reconfiguration, execution autonomy, and validation. Recurring patterns were grouped into informatics gaps, including one-way digital shadows, fragmented LIS/instrument semantics, single-case assumptions, weak command governance, and limited physical-actuation validation. This synthesis was interpretive and was not pre-registered, independently duplicated, or based on a validated requirement-elicitation instrument.

A proposed requirement was retained only if it addressed a documented gap and had either: (i) support from at least one direct pathology or clinical-lab source plus an independent source from another evidence tier, or (ii) support from at least two independent direct pathology or clinical-lab sources. Requirements were then restated without prescribing a technology. Where sources supported different implementations, the narrower common function was retained and the technologies were labelled as candidates; absence of direct pathology evidence was disclosed rather than bridged by adjacent-domain evidence. No minimum publication count was used beyond these rules, and no inference of superiority was made. The traceable chain was: taxonomy dimension evidence pattern capability gap implementation-neutral requirement candidate realization validation criterion. Dashboard-heavy D2/D3 findings motivated pre-action command governance; process-mining evidence motivated entity-aware state for decisions affected by derivative–object relationships; and cloud-dependent pathology AI motivated local deployment support only where latency, privacy, or scanner/LIS locality matter.

Each traceability claim was assigned to one or more evidence tiers: direct pathology evidence, clinical lab evidence, adjacent biomedical lab evidence, general methodological precedent, or an author-derived validation requirement. The first four tiers describe the provenance of cited evidence; the fifth identifies a proposed test or safeguard that remains to be evaluated. This hierarchy prevents robotics, self-driving-lab, and general process-method evidence from being represented as routine autonomous pathology deployment. Supplementary Fig. S1 reports the derivation workflow; the main text presents the architecture.

### Mapping corpus and validation resources

#### Analyzed mapping corpus

The evidence base comprises 74 database-derived primary records and 24 supplementary horizon-scanning records used for architectural design rather than field-frequency estimates. Each record contributed one D1–D4 tuple in Supplementary Table S1; no subsystem-level observation dataset was created. Supplementary Tables S1–S7 document records, exclusions, coding, traceability, denominators, methodological reference roles, and reviewer verification; Supplementary Fig. S1 reports architecture derivation. General methodological articles remained background references unless they addressed pathology or lab workflow systems with sufficient coding detail.

#### Denominator rules and interpretation boundary

Four denominators serve different purposes. The full evidence base is 98 records (74 primary plus 24 supplementary). Primary descriptive summaries and study-defined capability filters use 74 as their denominator. Fig. 2 uses 97 records because 1 record lacks a classifiable D3 value; Fig. 3 uses 93 because 5 records lack classifiable D1 values. Every included bibliographic record contributes at most one tuple to a figure. Counts including supplementary records describe the assembled evidence base and support architecture derivation; they are not estimates of prevalence in pathology practice or in all published literature.

**Fig. 2:**
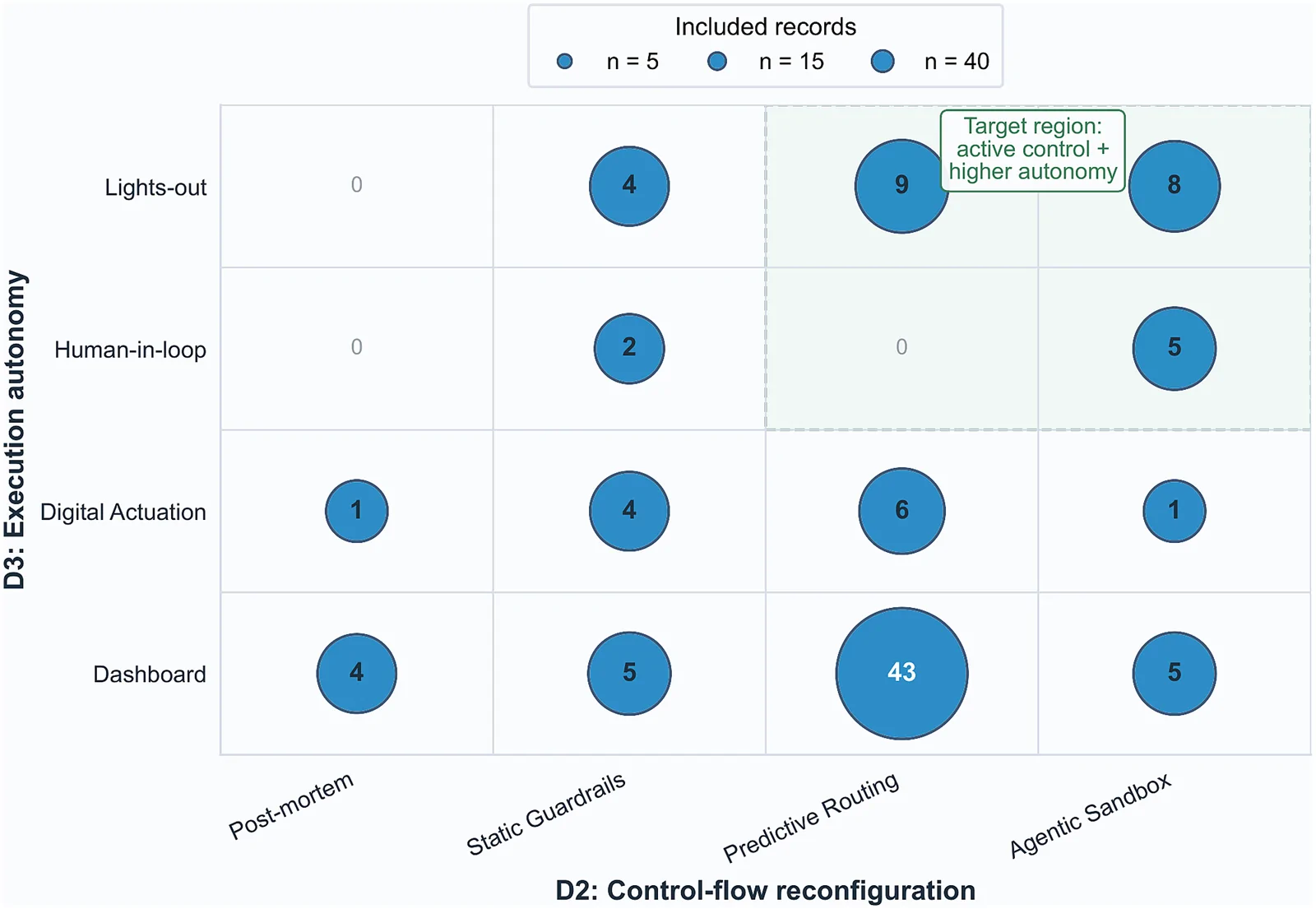
Evidence distribution across control-flow reconfiguration (D2) and execution autonomy (D3). Bubble area encodes 97 records with classifiable D2 and D3 codes; 1 record with insufficient D3 data is omitted. Primary and supplementary records are identified in Supplementary Table S1.

**Fig. 3:**
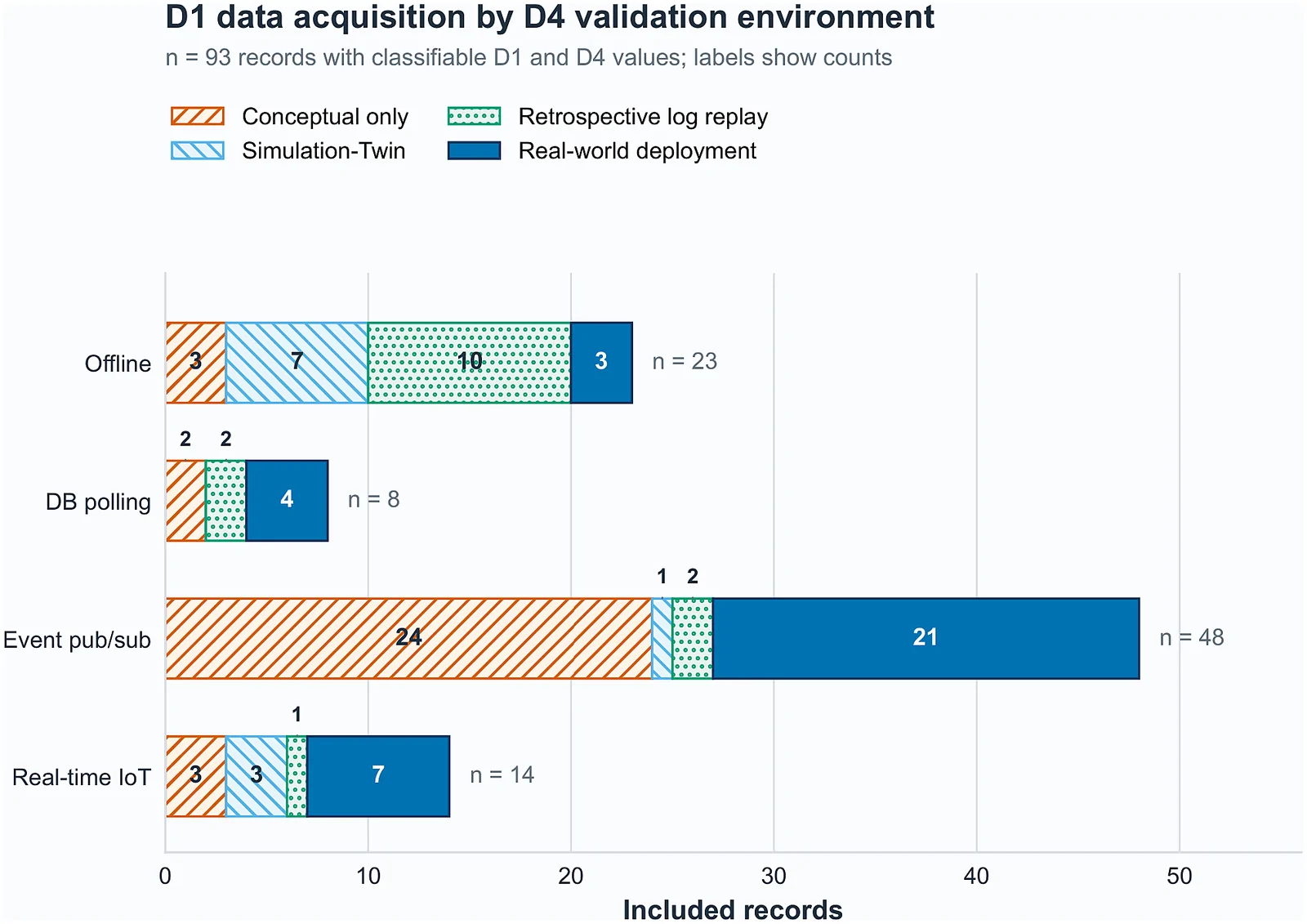
Validation environment (D4) by data-acquisition category (D1). Counts represent 93 records with classifiable D1 and D4 codes, each contributing one D1–D4 coding tuple; 5 records with insufficient D1 data are omitted. Primary and supplementary records are identified in Supplementary Table S1.

#### External validation resources

This study did not analyze an institutional clinical event log or prospective workflow-simulation trace, so external validation remains future work. Dataset choice must match the architecture layer being tested: whole-slide image datasets can support perception and image-routing evaluation, whereas event-log or electronic health record (EHR) resources can support process-simulation stress tests but cannot validate anatomical pathology image interpretation. Public pathology dataset benchmarking also depends on accessibility, provenance, annotation granularity, tissue coverage, staining, scanner metadata, and findable, accessible, interoperable, and reusable (FAIR) stewardship.^31^ Candidate substrates therefore have different evidentiary roles. CAMELYON can support perception generalizability, stain/scanner variation, and slide triage.^32^ PLCO pathology images can support provenance and domain-shift assessment.^33^ EHR or event-log resources such as MIMIC-IV can support synthetic event-log generation, queueing logic, conformance tests, and TAT metric design.^34^, ^35^ None can validate the full reference architecture alone.

Complete validation would require local pathology event logs covering accessioning, grossing, block creation, sectioning, staining, scanning, quality control, pathologist assignment, report authorization, instrument status, and exception handling. Image-level metrics alone would be insufficient. A complete evaluation would jointly report perception performance, process-mining conformance, median and tail TAT, queue length, resource utilization, unsafe-command rejection, human-override frequency, and latency from event ingestion to governed command output. Until such validation is performed, the architecture remains a derived design target.

## Results

### Temporal grounding of the evidence base

The evidence base spans 1993–2026, but the distribution is heavily weighted toward recent work. In the primary database corpus, 51 of 74 studies were published in 2023–2026 and 33 were published in 2025–2026. The supplementary horizon-scanning stratum is even more recent, with 21 of 24 records published in 2023–2026. This concentration supports treating agentic workflow orchestration, safety-sandbox governance, and real-time IoT-linked actuation as emerging evidence streams; the mapped corpus does not establish them as routine pathology lab practice.

The temporal profile of the coded dimensions is uneven rather than linear. Predictive routing and simulation precedents appear early in the corpus, including older capacity-planning and discrete-event simulation studies.^23^, ^24^ Physical actuation also has older robotic and rule-based automation precedents.^36^, ^37^ The D2 Level 4 agentic-sandbox category appears only in 2023–2026 in this corpus, whereas 7/8 primary D1 Level 4 records were published in 2023–2026. These sparse, corpus-specific signals indicate recent convergence of previously separate evidence streams; they do not establish a field-wide temporal transition or a deployment trend.

### Second-reviewer verification results

In the stratified 50-record independent blinded verification sample (30 included and 20 excluded full-text records), inclusion decisions agreed for 40/49 complete pairs (81.6%; unweighted κ=0.627). For mutually excluded records with complete categories, primary exclusion reasons agreed for 14/17 pairs (82.4%; unweighted κ=0.782). For mutually included records, 15 complete records contributed 60 D1–D4 dimension pairs. Exact agreement was 12/15 for D1 (80.0%; linearly weighted κ=0.842), 12/15 for D2 (80.0%; κ=0.909), 12/15 for D3 (80.0%; κ=0.837), and 12/15 for D4 (80.0%; κ=0.651). Overall field-level exact agreement across all evaluated dimensions was 48/60 (80.0%), which provided sample-based evidence of agreement, with variation across dimensions.

### Within-corpus operational distribution

The mapping reveals a capability gap between data availability, decision logic, execution autonomy, and validation setting. Fig. 2 maps the record-level D2 and D3 codes for 97 of 98 records with classifiable values in both dimensions; 1 record with insufficient D3 data is omitted from the grid. Primary and supplementary records remain traceable in Supplementary Table S1. The largest concentration appears in low-autonomy dashboard or advisory systems, especially systems that support predictive routing or retrospective process improvement without closing the actuation loop. The shaded upper-right quadrant marks the study-defined combination of active reconfiguration and high execution autonomy. Because supplementary agentic and robotic records contribute to this region, the figure is an evidence map for requirement derivation rather than an estimate of routine pathology deployment.

Fig. 3 shows a complementary pattern for the 93 records with classifiable D1 and D4 codes; 5 records with insufficient D1 data are omitted from the bars. Event publication and subscription are common among the record-level codes, but this does not automatically imply clinical readiness: many such records remain conceptual or depend on simulation rather than real-world deployment. Real-time IoT data links are less frequent, and only a subset are paired with real-world validation. Thus, the mapped evidence base contains many partial ingredients for pathology digital twins but few records that combine live data, autonomous control, physical execution, and deployment evidence.

At the record level, 8/74 primary records met the study-defined conjunctive high-capability filter (D1 ≥ 3, D2 ≥ 3, D3 ≥ 3, D4 ≥ 3), 2 met the operational closed-loop filter (D1 = 4 and D4 = 4 with D2 and D3 ≥ 3), and 1 met the full deployed-autonomy filter (D1 = D2 = D3 = D4 = 4). These are transparent within-corpus filters, not externally validated thresholds. The sole full-filter record was the adjacent biofilm-lab architecture reported by Gurung et al.^15^; its inclusion is not evidence of deployed autonomous pathology. Because qualifying records are heterogeneous, the counts show that many mapped records describe partial components and few records describe an integrated closed-loop system.

### The autonomy gap across the pathology ecosystem

The pathology ecosystem is constrained by structural complexity. Anatomical workflows split diagnostic material into specimens, blocks, slides, stains, scans, and reports, whereas core labs operate under high-volume TAT pressure. Core-lab process-improvement and workflow-tracing studies show that operational events can identify bottlenecks and support delay mitigation.^3^, ^4^, ^38^ This supports entity-aware state tracking and operational metrics for median, tail, and bottleneck behavior instead of dashboard averages alone.

Simulation, semantic-integration, and process-mining studies address different parts of the same gap. Discrete-event simulation and capacity-planning work established that lab routing can be modeled before operational change.^23^, ^24^ Process-mining and simulation studies extend this logic toward event-log replay and workflow discovery but often remain disconnected from physical actuation mechanisms.^6^, ^39^, ^40^ Lab AI, data harmonization, semantic workflow engines, and clinical decision-support add a complementary requirement: an integrated semantic-process layer that can align LIS, analyzer, scanner, report, and workflow events before orchestration is attempted.^41^, ^42^, ^43^

Agentic platforms, self-driving labs, robotics, and instrument-control studies connect digital decisions to protocol generation and physical interfaces.^44^, ^45^, ^46^ Pathology deployment, LIS-readiness, staff-readiness, and governance studies define clinical boundary conditions.^21^, ^47^ The derived requirement is bounded orchestration: command governance, scenario-based validation, and separation of related design evidence from direct pathology evidence.

### Mapping-derived reference architecture

The reference architecture follows from the Architecture Derivation Method and the traceability chain summarized in Table 4. It organizes six functional areas for staged evaluation: the operational pathology environment, semantic perception and shared state, entity-aware process representation, bounded orchestration, pre-action command governance, and governed actuation with a human authority boundary; local execution support is conditional on deployment constraints. Fig. 4 depicts a safety-and-constraints sandbox as one candidate realization of pre-action governance, not as an invariant architecture requirement. Specific formalisms and orchestration technologies remain candidates for comparative evaluation.

**Table 4:** Condensed evidence-to-architecture traceability. Full study-level links, evidence tiers, and derivation notes are provided in Supplementary Table S3.

ID	Mapping finding	Derived architecture response	Evidence hierarchy	Required validation
T01	Monitoring-heavy digital shadows^3^, ^4^; adjacent-domain pre-action safety filtering^15^	Pre-action command governance; candidate realization: safety-and-constraints sandbox	Clinical lab; adjacent biomedical lab; author-derived validation	Command rejection; pre-action simulation; override logging
T02	Case-to-specimen-to-slide entity fission^5^, ^6^, ^12^	Entity-aware event layer plus candidate OCPN/CPN formalism	Direct pathology; general methodological precedent; author-derived validation	Multi-entity trace replay; object-link consistency
T03	Heterogeneous LIS, analyzer, scanner, report, and local-code events^41^, ^42^, ^43^	Semantic event and provenance contract; FHIR, DICOM, SiLA 2, and OCEL are candidate bindings by interface	Clinical lab; author-derived validation	Profile/conformance checks; identifier binding; local-code mapping; event alignment
T04	Robotics, MAS scheduling, and command-interface precedents^14^, ^25^, ^45^, ^46^	Governed coordination and command interface; rules, BPMN, optimization, and MAS are candidates	Direct pathology; adjacent biomedical lab; general methodological precedent; author-derived validation	Scenario testing; exception recovery; command provenance; fail-safe behavior
T05	Cloud-dependent pathology AI deployment friction^20^, ^21^	Conditional edge/local inference and trace buffering	Direct pathology; author-derived validation	Latency and privacy assessment; buffer and fail-safe tests
T06	Right-skewed TAT and bottleneck behavior^3^, ^4^, ^23^	Robust operational metrics in the validation agenda	Clinical lab; author-derived validation	Median/IQR TAT; tail delays; queue length; utilization

**Fig. 4:**
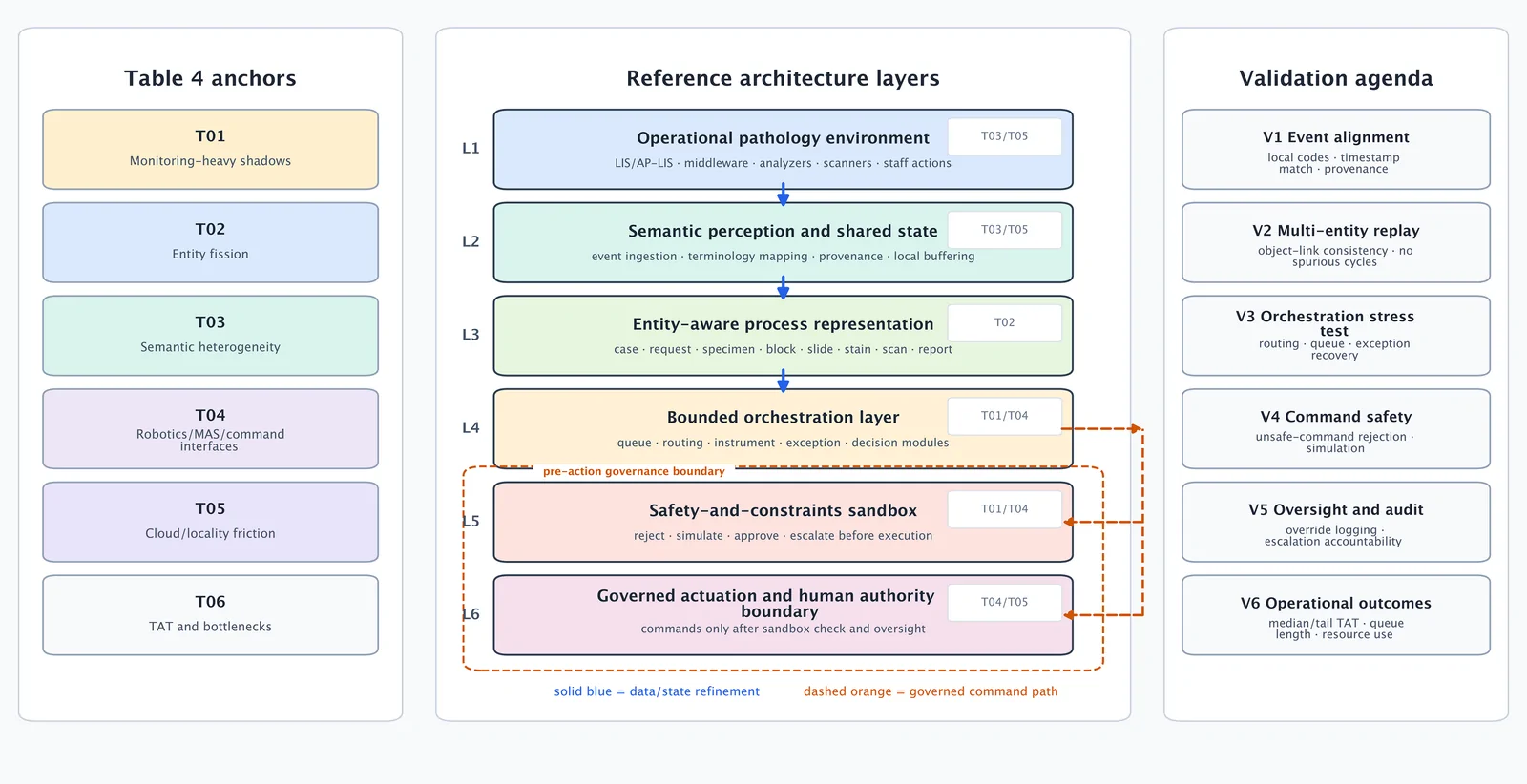
Evidence-derived reference architecture for entity-aware pathology process informatics. Solid blue arrows indicate data/state refinement from operational events through semantic state and entity-aware representation toward bounded orchestration. Dashed orange arrows indicate a governed command path through a candidate safety-and-constraints sandbox to governed actuation. The invariant requirement is pre-action command governance; displayed components are candidate interfaces, not an implemented or validated system. (For interpretation of the references to color in this figure legend, the reader is referred to the web version of this article.)

#### Perception layer: Semantic state reconstruction

Pathology orchestration requires reconstruction of operational state across LIS records, middleware events, analyzers, stainers, scanners, images, and reports. The informatics gap is not vocabulary mapping alone; it is cross-system event alignment, provenance preservation, and semantic state reconstruction for entities that move across local instrument and reporting systems. Lab measurement and data-warehouse studies show why this matters: identical analytes can diverge across instrument platforms, and a single data-warehouse query may return hundreds of local labels that do not map cleanly to the intended clinical concept.^41^, ^42^

Interoperability standards respond to this gap but do not remove local variation. Their roles are complementary. HL7 FHIR can exchange orders, tasks, specimens, observations, and diagnostic reports, but does not by itself encode every local case–block–slide relationship or an instrument command policy.^48^ DICOM whole-slide imaging and the DICOM specimen module connect images to specimen identification and preparation, but do not provide AP-LIS scheduling or enterprise orchestration.^49^ SiLA 2 exposes lab-device features, properties, and commands, but does not supply clinical semantics or authorization boundaries. OCEL 2.0 represents event-to-object and object-to-object relationships for analysis and exchange, but is not a clinical terminology or device-control standard. SNOMED CT, LOINC, UCUM, RDF/OWL representations, and graph databases provide further building blocks for semantic agreement and provenance-aware traversal.^19^, ^43^, ^45^, ^50^ A deployable state layer must profile and bind these standards to local identifiers, workflow states, and governance rules rather than treating standards compliance as plug-and-play interoperability.

Beyond structured fields, unstructured reports may require natural language processing when free text affects state reconstruction, handoff interpretation, or quality-control routing. Healthcare knowledge-graph reviews further indicate that semantic middleware remains constrained by data quality, interoperability, and deployment barriers.^51^ The validation of the perception layer therefore prioritizes cross-system event alignment, local-code reconciliation, and provenance completeness.

#### Entity-aware representation and bounded orchestration layer

This layer has two linked responsibilities: representing derivative pathology entities and coordinating bounded actions over that representation. A monolithic central controller may have difficulty with this task because a single surgical resection case generates one request form, multiple specimens, paraffin blocks, and multiple glass slides. This one-to-many hierarchical fission means that identifiers, routing states, and processing statuses must be tracked simultaneously and independently.

The core requirement is therefore an object-centric or entity-aware operational state model. OCEL-like event models provide one candidate event-log layer because they allow events to be linked to multiple objects and object types rather than flattened into a single case view.^11^, ^19^ OCPNs extend this logic toward process discovery and replay by associating places with object types and transitions with collections of typed objects.^12^ In pathology terms, these objects may include request forms, specimens, blocks, slides, stains, scans, reports, and instrument or worklist states.

The pathology-specific evidence supports this requirement but does not uniquely select one formalism. Stünkel et al. identify hierarchical case-to-slide fission as a defining obstacle for standard process mining tools in digital pathology.^6^ When they applied conventional process discovery algorithms from the ProM software suite to real lab event logs, most algorithms produced models where all activities could occur in parallel, and spurious cycles appeared on every activity node. These artifacts arose because the algorithms could not model different types of tokens flowing through the net when a case splits into blocks and slides. CPNs are therefore a useful simulation-oriented candidate because token colors can represent entity classes, but OCPN and artifact-centric workflows are also relevant alternatives for object lifecycles and inter-object interactions.^12^, ^13^

Dynamic routing also requires coordination logic over the selected representation. Lee and Yang address this complementary requirement through a MAS architecture with job, material-handling, machine, and queue agents communicating through message-passing interfaces.^25^ Their state-action diagrams show how distributed agents can respond to local perturbations without global replanning. In pathology, this is a candidate rationale, not an evidence that MAS is superior to a rule engine or centralized optimizer; the appropriate choice depends on state quality, perturbation frequency, verifiability, and command risk.

Pańkowska et al. apply a comparable digital-twin logic to the pathomorphological diagnosis process.^14^ They construct an IoTDT-BPMN layer that encodes workflow logic as a BPMN specification, then use a digital-twin environment to generate agents and simulate process execution. The contribution for the present architecture is the explicit, inspectable coupling between process logic and agent behavior; it does not establish multi-site deployment robustness.

This layer therefore does not prescribe CPN, OCPN, BPMN, or MAS. It specifies a representation-and-coordination contract: an OCEL-like event layer or equivalent object-centric log should preserve entity links; candidate executable formalisms should support replay or scenario testing; and any rule engine, optimizer, workflow engine, or MAS coordinator should read the same governed state and emit commands through the same validation gateway. Comparative implementation should evaluate model fitness, explainability, failure recovery, computational latency, and unsafe-command rejection rather than selecting a formalism by analogy alone.

#### Actuation and local execution layer: Governed command interfaces

Digital scheduling decisions acquire operational value only when they can be translated into verifiable physical or digital actions within local pathology constraints. In core labs, commands may affect analyzers, robotic tracks, centrifuges, or middleware queues; in anatomical pathology, they may affect stainers, scanners, AP-LIS states, slide-routing worklists, or instrument-local buffers. This requirement defines the future actuation layer: sandbox-approved commands must remain compatible with local device interfaces, middleware permissions, cybersecurity rules, command provenance, and human-override boundaries. SiLA 2 and robot operating system (ROS) provide relevant design precedents for instrument control and robotic middleware.

Juchli establishes the command-interface problem addressed by SiLA 2: heterogeneous instruments, vendors, software packages, and protocols require more than file exchange.^45^ SiLA 2 defines instrument features through properties, commands, and SiLA data types, with transport-layer security by default. For pathology orchestration, the requirement is a uniform, auditable vocabulary through which a governed controller can issue execution commands to compliant devices; the controller need not be an agent.

Zsoldos et al. provide an implementation example using ROS as robotic middleware.^52^ Their hierarchy decomposes lab workflows into tasks, subtasks, and low-level robotic activity representations, whereas integration problems across ROS, Python, operating systems, and robot firmware show the need for explicit version governance.

Knobbe et al. add the physical command-interface perspective: telemetry, digital-twin synchronization, and precision validation can be coupled to robotic liquid handling.^46^ These studies identify explicit command-interface and middleware contracts as necessary when evaluating physical actuation in closed-loop pathology workflows; they do not imply that every monitoring or scheduling study requires device control.

The actuation layer therefore operates on two physical scales. At the instrument scale, SiLA 2-like command interfaces could support governed interactions with analyzers, centrifuges, staining platforms, scanners, or other devices. At the logistics scale, ROS-like middleware and collaborative robotics could support transport or robotic handling when such platforms are locally available.^53^ These precedents specify command-interface and local-execution requirements for future validation: latency measurement, unsafe-command rejection, human-override logging, cybersecurity review, command provenance, and fail-safe behavior.

#### Governance layer: Safety-and-constraints sandbox

Clinical lab environments impose safety and compliance requirements that constrain autonomous execution. A controller that issues physically irreversible commands without prior validation poses unacceptable risk. The implementation-neutral requirement is a pre-action governance function that checks proposed routing strategies against clinical, operational, and technical rules and produces an explicit command class: reject, simulate, approve, or escalate. The safety-and-constraints sandbox shown in Fig. 4 is an author-defined candidate realization of that function, not a validated component or digital twin.

Gurung et al. provide a close adjacent-domain precedent within the mapped corpus.^15^ Their autonomous biofilm-engineering architecture includes a component named the *Safety and Constraints Twin*, which encodes biosafety, regulatory, and operational constraints and filters actions proposed by a control twin before execution. The candidate sandbox adapts that general pre-action-filtering pattern to pathology; it does not claim to originate or validate the source concept.

Rahimi et al. place this requirement within responsible AI-enabled digital twin development, emphasizing that reliability, safety, transparency, and accountability must be embedded before deployment.^16^ In the reference architecture, pre-execution simulation and constraint checking make these concerns testable at the command boundary.

Monek and Fischer provide adjacent-domain scheduling evidence for pre-execution filtering.^54^ Their expert twin uses a threshold-based fuzzy-logic module to decide whether an optimization gain justifies rescheduling. In this review, that result informs command filtering and anti-jitter logic instead of clinical pathology readiness.

The governance layer therefore specifies a staged command protocol: orchestration proposes a routing strategy; the sandbox simulates it against active compliance rules; and the sandbox rejects, holds for further simulation, approves for bounded execution, or escalates to a human operator. This preserves potential scheduling speed without bypassing clinical pathology regulation.

#### Evidence-to-architecture traceability

Table 4 summarizes the evidence-to-architecture traceability logic used to derive the components visualized in Fig. 4. Detailed mapping links and supporting evidence are provided in Supplementary Table S3.

## Discussion

### Implications for pathology informatics practice

The mapping places implementation responsibility across the pathology ecosystem rather than in a single model. Lab and anatomical pathology information system teams need stable identifiers and relationships across case, specimen, block, slide, stain, scan, and report objects. Instrument, scanner, middleware, and AP-LIS/LIS interfaces need timestamped events with source, transformation, and command provenance. Without these foundations, queue predictions or agent recommendations cannot be traced back to the physical and digital states that produced them.

For vendors and lab IT teams, the immediate design target is an auditable event and command contract: which system observed an event, which object changed, which rule or model proposed an action, and which interface accepted, rejected, or escalated it. Workflow managers can use the same representation for bottleneck and exception analysis before introducing actuation. Pathologists and lab staff remain the authority for diagnostic judgment, report authorization, and exceptions, whose clinical meaning exceeds bounded operational rules. Any automated routing or instrument assignment should preserve human override and produce a reviewable action history.

Implementation should therefore proceed in phases. A lab can first validate event completeness and object links, then replay historical traces, test proposed commands in a sandbox, conduct human-supervised shadow operation, and only then evaluate narrowly bounded actuation prospectively. This progression matches recent pathology-informatics emphasis on interoperability, implementation context, and governance while avoiding the inference that a reference architecture alone establishes clinical readiness.^8^, ^9^, ^10^

### Pathology scenarios, variability, and failure recovery

The architecture can be interpreted through five practical scenarios. These are design-test specifications derived from pathology workflow constraints, not completed implementations; site-specific thresholds would need approval before prospective use.

• *Frozen section:* A time-critical specimen, block, slide, image, and preliminary interpretation must remain linked while priority changes. The controller may propose queue escalation or scanner assignment, but tissue handling and diagnostic release remain under named human authority. *Fallback/test:* Revert to the approved manual priority pathway if any entity link or acknowledgement is missing; pass only if every derivative object remains traceable and no diagnostic action is automated.
• *Rescan after image-quality failure:* A scanner quality-control event should link the failed scan, physical slide, replacement scan, and worklist status. A bounded digital command may create a rescan task; it must not silently replace the image associated with an authorized report. *Fallback/test:* Hold both images for human reconciliation; pass only if the failed scan remains auditable and the replacement cannot alter an authorized report without approval.
• *Recut or additional stain:* A pathologist-authorized request creates new derivative slide entities and resource dependencies. The state model must prevent duplicate orders and tissue-exhaustion risks; no new tissue-processing command should proceed without the required authorization. *Fallback/test:* Stop at a manual worklist when authorization, tissue sufficiency, or object links are uncertain; pass only if duplicate and unauthorized commands are rejected.
• *Instrument failure:* A stainer, scanner, or analyzer outage requires reconciliation of in-flight entities, stale events, and local buffers before rerouting. The safe response is to suspend affected commands, expose uncertainty in current location/state, and use an approved fallback route rather than infer successful completion. *Fallback/test:* Require reconciliation before restart; pass only if in-flight entities are accounted for and stale or duplicate acknowledgements cannot trigger execution.
• *Priority oncology case:* Urgency can alter queue position only within explicit service, fairness, and specimen-readiness constraints. The decision record should retain the triggering evidence, displaced work, override authority, and downstream TAT effect. *Fallback/test:* Preserve the existing queue or escalate to a workflow manager when constraints conflict; pass only if the priority decision and displaced work are reviewable and policy-conformant.

Generalizability is therefore conditional on local configuration. Staining protocols, scanner optics and file formats, AP-LIS/LIS data models, middleware behavior, staffing, resource capacities, accessioning conventions, and institutional authority policies can all change both the observed state and the set of permissible commands. A deployable implementation would require site-specific profiles and cross-site tests rather than assuming that an architecture configuration transfers unchanged across labs.

Process-level robustness should be evaluated separately from predictive-model robustness. Event tests should inject dropped, duplicated, delayed, and out-of-order messages; broken case–specimen–block–slide links; clock drift; unavailable instruments; stale worklists; and partial command acknowledgement. Minimum reporting should include event completeness, entity-link error rate, timestamp discrepancy, state-reconciliation time, command latency, rejected unsafe commands, fallback success, human overrides, and recovery time. A controller should enter a defined safe state when evidence is incomplete rather than convert missingness into a confident route.

Where an architecture component uses machine learning, discrimination alone is insufficient. Digital-pathology studies show that scanner and staining variation can create domain shift, and that uncertainty estimates can support abstention or OOD detection, although performance must be validated for the intended deployment distribution.^55^, ^56^, ^57^ Evaluation should stratify performance by institution, scanner, stain, specimen type, and workflow phase; report calibration and uncertainty; test near- and far-out-of-distribution inputs and domain shift; define abstention and escalation thresholds; and monitor drift after deployment. Useful measures include calibration plots, Brier score or expected calibration error, risk–coverage curves for selective prediction, OOD detection performance, and the proportion and outcomes of abstained cases. Measurement traceability, calibration, and reproducibility across the wider digital-pathology pipeline should be assessed alongside model metrics.^58^ These metrics govern model-assisted routing or triage; they do not transfer diagnostic authority to the model.

### Pathology digital twins require more than bidirectional data flow

The mapping results show that bidirectional synchronization alone is insufficient for pathology workflow digital twins. Under the operational criterion adopted here, a workflow twin should represent relevant workflow entities, support auditable intervention logic, and connect proposed interventions to governed physical or digital actions. Systems that extract data and visualize delays without these capabilities are better understood as digital shadows, even when they provide high-fidelity synchronization. This distinction matters because over-labeling dashboards or standalone models as digital twins can obscure the methodological work still required: event semantics, entity-aware state, actuation readiness, command governance, and validation criteria.

This boundary is illustrated by two complementary evidence streams. Sadée et al. and Monek and Fischer show why synchronization or model fidelity does not by itself establish operational control,^17^, ^54^ whereas Cai et al. demonstrate a disciplined digital-shadow framing for timestamp extraction and workflow improvement without claiming physical actuation.^4^ Future empirical studies should therefore report data-flow directionality, entity representation, actuation capability, and safety constraints as minimum disclosure items.

### Entity-aware representation for multi-entity pathology decisions

The central implication is conditional: entity-aware process representation is important when a pathology decision depends on clinically coupled but operationally asynchronous specimens, blocks, slides, stains, scans, reports, or amendments. Core labs demonstrate high-volume operational pressure and TAT sensitivity, whereas anatomical pathology exposes this multi-entity representation problem directly. A case-centric or centralized controller may remain sufficient for bounded tasks whose state and authority can be represented without derivative-object dependencies; it becomes inadequate when those dependencies materially change the inferred state or permissible action.

OCPM provides an established methodological foundation for this issue. OCEL and OCPM address the case-centric limitation by allowing events to relate to multiple objects and object types, and healthcare research has shown how data models such as the observational medical outcomes partnership can be transformed into object-centric event logs.^7^, ^11^, ^18^ The pathology-informatics challenge is to integrate such representation with lab semantics, digital-twin synchronization, governed actuation, and validation criteria.

Stünkel et al. identify this limitation at the level of pathology process-mining method.^6^ Standard process discovery algorithms produced spurious cycles in pathology event logs because they could not distinguish multiple simultaneous token streams generated from one case root. CPNs provide a defensible representation example because token colors can distinguish request forms, specimens, blocks, and slides while preserving their shared case context. OCPNs and artifact-centric workflows provide related alternatives for multi-object process behavior.^12^, ^13^ The resulting claim is not that CPNs alone solve pathology automation, but that multi-entity relationships must be represented explicitly before evaluating scheduling, predictive routing, or actuation decisions whose validity depends on those relationships.

The publication-year profile supports treating the architecture as a convergence framework instead of a mature deployed stack. Earlier studies established simulation, capacity planning, rule-based support, and robotic automation precedents.^23^, ^24^, ^36^ Recent work adds semantic interoperability, pathology process mining, robotic digital twins, agentic workflows, clinical AI governance, and safety-constrained autonomy.^6^, ^15^, ^16^, ^25^, ^45^

Evaluation methods must also match this process-informatics framing. TAT distributions in clinical labs are typically right-skewed, and tail delays often carry more operational significance than mean delay. Cai et al. and Park et al. illustrate non-parametric handling of TAT distributions, including tests for non-normality and median/tail-focused reporting.^3^, ^4^ Carobene et al. further show why lab data require careful characterization of methods, units, traceability, and analytical platforms.^42^ Future pathology orchestration studies should evaluate both process-model validity and operational performance: conformance fitness, event completeness, tail TAT, queue length, unsafe-command rejection, human-override frequency, and latency from event ingestion to validated action.

### Closed-loop orchestration requires staged validation before autonomy claims

The architecture allows MAS, a safety sandbox, edge deployment, and bounded large language model (LLM) interfaces as candidate implementation choices. MAS may support distributed exception handling when a centralized scheduler cannot respond robustly to local perturbations, but deterministic rules, BPMN engines, and optimization remain preferable where they are simpler to verify. Robotics and self-driving lab studies inform the design; pathology-specific readiness still depends on staged validation in routine lab settings.

Pre-action command governance is required before evaluating bounded autonomy. Gurung et al. describe a *Safety and Constraints Twin* that filters proposed actions through biosafety, regulatory, and operational constraints before execution^15^; Rahimi et al. situate this requirement within responsible AI-enabled digital twin development.^16^ The present architecture depicts a safety-and-constraints sandbox as one candidate pathology gateway that could reject unsafe commands, simulate routing consequences, document human overrides, and define escalation boundaries.

Local edge deployment is similarly conditional instead of universal. Angeloni et al. and Deman et al. show that cloud-dependent pathology AI can create integration, privacy, and operational constraints, especially when whole-slide images, proprietary viewers, AP-LIS connections, VPNs, or IP whitelisting are involved.^20^, ^21^ Edge or institutional deployment is justified when latency, scanner/LIS locality, trace buffering, privacy, or governance constraints materially affect orchestration. LLM components should remain limited to bounded interface, explanation, or protocol-interpretation roles; deterministic routing logic and numeric control should remain in auditable modules and pass through an approved pre-action governance gateway before any command is executed.

The human boundary remains central. Gruson et al. describe a progression from traditional automation through conventional AI to agentic AI while retaining human responsibility.^47^ Eccher et al. similarly frame digital twins as supporting selected workflow decisions while preserving final diagnostic authority.^22^ The appropriate boundary is staged autonomy: low-risk logistics, routing, instrument assignment, priority escalation, and quality-control flagging may become candidates for bounded automation, whereas diagnosis, therapeutic implication statements, and report sign-off remain human clinical responsibilities.

Validation resources follow the same staged logic. Public whole-slide image datasets can support evaluation of perception-layer generalizability and domain shift, whereas EHR/event-log resources can support synthetic event-log generation, conformance testing, queueing logic, and tail-delay metrics. Neither class of resource validates the full architecture alone. This is a validation agenda rather than a preset roadmap: before each site-specific stage, investigators must prospectively define the baseline, entry criteria, acceptable error and unsafe-command rates, human-override authority, rollback trigger, and exit criterion. Advancement from trace validation to replay, shadow operation, or bounded actuation should occur only after the prior gate is met; otherwise the system returns to the last approved manual or shadow mode. A credible next-stage study would combine a local pathology event log, a synthetic multi-entity trace replay, expert review of command boundaries, gateway rejection tests, cybersecurity assessment, and prospective workflow evaluation before claiming operational readiness.

### Limitations

First, this is structured evidence mapping with targeted horizon scanning, not a reproducible comprehensive systematic review of every pathology automation intervention, and searches were restricted to English. The purposive Google Scholar component captured emerging agentic-science systems as design evidence, not field-frequency evidence. Its result order, number screened, and deduplication trail were not retained; citation-tracking seeds, rounds, and stopping criteria were likewise not preserved. The historical PubMed field-tag syntax, IEEE Xplore interface settings, and original export files are also unavailable. Reconstructed rerun translations are disclosed in supplementary search strategies but cannot substitute for the original search history. This incompleteness constrains the framing of the entire evidence map as a dated corpus snapshot and may have affected which records were retrieved. The review was not prospectively registered and no separate protocol was prepared.

Second, full-corpus screening and initial extraction were conducted by a single investigator. To evaluate coding reproducibility, an independent blinded verification was conducted on a stratified 50-record sample, yielding 81.6% inclusion agreement (κ=0.627) and 80.0% overall D1–D4 field agreement (κ=0.651–0.909 across dimensions). While providing sample-based evidence of consistency, the verification is sample-based and cannot fully substitute for prospective double-screening across the entire retrieval output. Furthermore, the D1–D4 operational capability taxonomy is an author-developed analytical framework rather than an internationally standardized validation instrument. Consequently, the mapped distributions should be interpreted as descriptive evidence patterns within the assembled corpus rather than epidemiological prevalence estimates of lab automation across all pathology centers.

Third, this descriptive mapping did not include a formal risk-of-bias assessment or certainty grading, and the purposive supplementary stratum should not be used for frequency estimates. Fourth, several sources come from related domains, including manufacturing digital twins, self-driving labs, robotics, OCPM, and healthcare digital-twin reviews. These sources support requirements or candidate implementation patterns, not routine pathology deployment. Fifth, the reference architecture is a synthesis of mapped requirements rather than a deployed system, expert-validated framework, or prospective implementation. It does not establish that MAS, OCEL, OCPNs, CPNs, artifact-centric workflows, BPMN, edge deployment, or the safety-and-constraints sandbox are superior for pathology. Prospective comparison must include expert review, site-specific trace construction, synthetic multi-entity replay, missing-event and instrument-failure stress tests, process simulation, command-rejection tests, cybersecurity assessment, calibration and OOD evaluation for AI components, and staged clinical implementation.

## Conclusion

Pathology automation cannot be evaluated solely by dashboard, image-analysis, or middleware sophistication. The central pathology-informatics challenge is entity-aware process representation and governed orchestration under operational constraints. Within this structured 98-record map, many records addressed partial capabilities and few records described a combination of live sensing, entity-aware process state, bounded reconfiguration, governed actuation, and real-world validation. The corpus and its single-reviewer origins do not justify a field-level prevalence claim. The same requirements may inform other multi-entity biomedical workflows, but transfer requires separate evaluation.

The reference architecture is a mapping-derived design target for staged validation. It connects semantic perception, entity-aware process state where multi-entity dependencies matter, implementation-neutral bounded orchestration, pre-action command governance, governed actuation, and conditional local deployment. A safety-and-constraints sandbox, rule engines, BPMN, optimization, MAS, OCPN, and CPN remain candidate realizations rather than validated choices. The next step is comparative evaluation using pathology event traces, failure injection, process replay, operational and AI-robustness metrics, cybersecurity tests, expert review, and prospective workflow assessment while preserving the boundary between bounded logistics and diagnostic authority.

## CRediT authorship contribution statement

**Ao Li:** Conceptualization, Methodology, Software, Formal analysis, Investigation, Writing – Original Draft, Writing – Review & Editing, Visualization. **Peter O'Loughlin:** Conceptualization, Methodology, Validation, Writing – Review & Editing. **Lin Yue:** Writing – Review & Editing. **Wei Emma Zhang:** Writing – Review & Editing. **Bernadette Swart:** Writing – Review & Editing. **Weitong Chen:** Conceptualization, Methodology, Validation, Writing – Review & Editing, Supervision.

## Ethics statement

This review analyzed published literature and bibliographic metadata only. It involved no research participants, patient-level data, or protected health information; institutional ethics approval was not required.

## Funding

This work was supported by 10.13039/501100000923Australian Research Council ARC Early Career Industry Fellowship (Grant No. IE240100275), Australian Research Council Discovery Projects (Grant No. DP240103070), Australian Research Council Linkage (Grant No. LP230200821), and Australia's Economic Accelerator Ignite (Grant No. IG250200014).

## Declaration of generative AI use

During the preparation of this manuscript, the authors used OpenAI's ChatGPT/Codex for language and readability support, document organization, computational and file-consistency checks, and text-only editing of the graphical abstract. The tools did not make literature-screening, inclusion, D1–D4 coding, adjudication, or second-reviewer decisions. The authors verified the affected text, calculations, citations, and visual content and take full responsibility for the article.

## Declaration of competing interest

The authors declare that they have no known competing financial interests or personal relationships that could have appeared to influence the work reported in this article.

## Figures and Tables

**Supplementary Figure S1:**
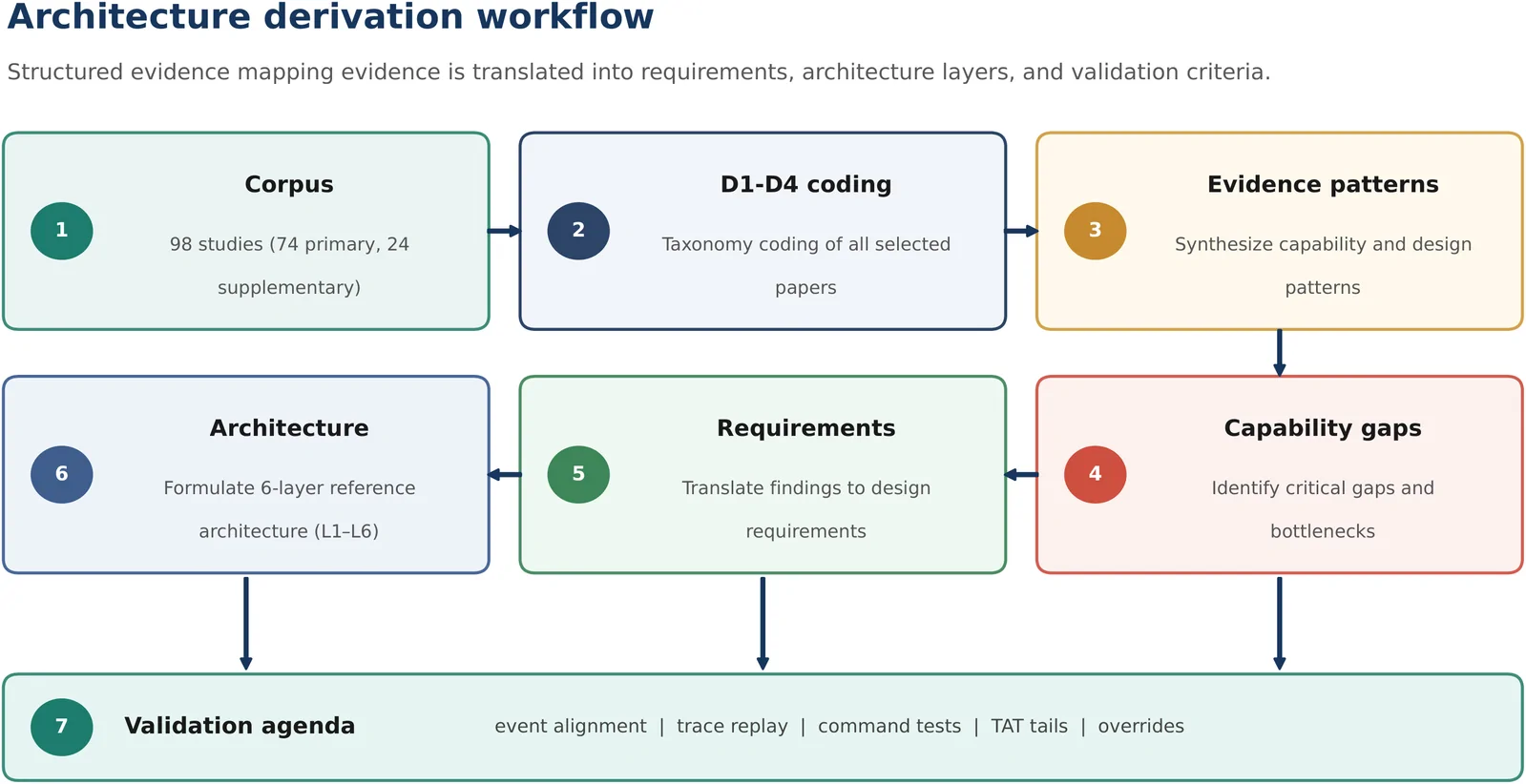
Workflow for deriving architecture requirements from the structured evidence map, evidence patterns, capability gaps, and validation criteria.

## Data Availability

The supplementary files contain the record-level mapping data, coding rules, denominator guide, and anonymized reviewer-verification summary. The version-controlled repository is https://github.com/AoXan/pathology-workflow-informatics-systematic-mapping. It is private during double-anonymized review; editorial access can be provided on request.

## Appendix A Supplementary data

Supplementary Table S1 Complete 98-record evidence base, including 74 primary database records and 24 supplementary horizon-scanning records. Each bibliographic record contributes one D1-D4 coding tuple.

Supplementary Table S2 Record-level log for 124 records excluded at full-text eligibility assessment, with standardized primary exclusion reasons.

Supplementary Table S3 Evidence-to-requirement traceability matrix distinguishing direct pathology, clinical laboratory, adjacent biomedical laboratory, general methodological, and author-derived validation evidence.

Supplementary Table S4 Record-level denominator guide for manuscript displays, including Fig. 2 (97 classifiable D2-D3 records) and Fig. 3 (93 classifiable D1-D4 records).

Supplementary Table S5 Background methodological literature and standards used to interpret process representation, interoperability, governance, and validation.

Supplementary Table S6 Author-developed D1-D4 coding definitions, boundary rules, and representative examples used for record-level coding and reviewer verification.

Supplementary Table S7 Methodological assessment of the historical 30-record visible-code verification, the blinded design of the 50-record exercise, and the final comparison after second-reviewer re-examination, including agreement metrics and an anonymized classification trace. Private reviewer comments and internal discussion records are not included.

Supplementary material 1 Supplementary Search Strategies. Retained historical search information and explicitly labelled current rerun translations, including unrecoverable search-history details.
