# Supplementary material for: Entity-aware pathology workflow informatics: Structured evidence mapping and a reference architecture for governed orchestration
Source: J Pathol Inform. 2026 Sep 14;23:100720. doi: 10.1016/j.jpi.2026.100720 (PMC13676132; doi:10.1016/j.jpi.2026.100720)
Supplement: Supplementary file 1 [file mmc9.docx]

**Supplementary File S5: Comprehensive Search Strategies and Rerun Protocols**

*Journal of Pathology Informatics | Manuscript: JPATHINFORMATICS-D-26-00119*

**1. Search Overview and Methodological Statement**

This document details the literature search strategies, boolean syntax, conceptual query building blocks, and platform-specific rerun protocols for the structured evidence mapping review. All primary database and supplementary searches were conducted on May 1, 2026.

*PRISMA-S Transparency Declaration: To adhere strictly to PRISMA 2020 and PRISMA-S reporting recommendations, this document transparently distinguishes between: (i) exact contemporaneously preserved syntax (Scopus), (ii) conceptual query blocks with platform-specific rerun adaptations (PubMed and IEEE Xplore), and (iii) targeted horizon-scanning discovery protocols (Google Scholar and citation tracking). Reconstructed or adapted syntax is explicitly identified as such.*

**2. Multi-Database Retrieval Summary (Searched May 1, 2026)**

| **Information Source** | **Search Date** | **Initial Retrieval (n)** | **Syntax / Reproducibility Status** |
| --- | --- | --- | --- |
| Scopus (Elsevier) | 2026-05-01 | 349 | Exact retained executable query syntax |
| PubMed (NCBI / NLM) | 2026-05-01 | 31 | Conceptual expression retained; current rerun translation provided |
| IEEE Xplore | 2026-05-01 | 11 | Conceptual expression retained; current rerun translation provided |
| Google Scholar | 2026-05-01 | 10 (retained) | Targeted horizon scan for emerging agentic / self-driving laboratory systems |
| Citation Tracking | 2026-05-01 | 14 (retained) | Backward and forward citation tracking of seed pathology workflow literature |

**3. Detailed Boolean Search Strategies by Database**

**3.1. Scopus (Elsevier)**

• Search Date: May 1, 2026
• Field: TITLE-ABS-KEY
• Language Limit: English
• Hits: 349 records

```
Scopus Retained Historical Executable Query:
TITLE-ABS-KEY(
    (( "digital twin*" OR "digital shadow*" OR "virtual twin*" OR "cyber-physical system*" )
     AND ( "pathology lab*" OR "clinical lab*" OR "core lab*" OR "medical lab*" OR "laboratory medicine" ))
    OR
    (( "artificial intelligence" OR "machine learning" OR "process mining" OR "discrete event simulation" OR "ontology" OR "knowledge graph" )
     AND ( "pathology lab*" OR "clinical lab*" OR "core lab*" OR "medical lab*" OR "laboratory medicine" )
     AND ( "workflow*" OR "turnaround time" OR "TAT" OR "throughput" OR "bottleneck*" OR "scheduling" OR "process improvement" ))
)
```

**3.2. PubMed (National Center for Biotechnology Information / NLM)**

• Search Date: May 1, 2026
• Interface: PubMed Advanced Search
• Historical Retrieval: 31 records
• Status: Conceptual terms preserved; standardized field-tagged rerun translation verified via NCBI E-utilities API.

**PubMed Standardized Rerun Translation:**
(( "digital twin"[All Fields] OR "digital twins"[All Fields] OR "digital shadow"[All Fields] OR "virtual twin"[All Fields] OR "cyber-physical system"[All Fields] )
 AND
 ( "pathology laboratory"[All Fields] OR "clinical laboratory"[All Fields] OR "core laboratory"[All Fields] OR "medical laboratory"[All Fields] OR "laboratory medicine"[All Fields] OR "clinical lab"[All Fields] OR "lab automation"[All Fields] ))
AND english[Language]

**3.3. IEEE Xplore Digital Library**

• Search Date: May 1, 2026
• Interface: IEEE Xplore Advanced Search
• Historical Retrieval: 11 records
• Status: Conceptual terms preserved; mapped to 'All Metadata' query structure.

**IEEE Xplore Advanced Search Translation:**
(( "All Metadata":"digital twin" OR "All Metadata":"digital twins" OR "All Metadata":"digital shadow" OR "All Metadata":"virtual twin" OR "All Metadata":"cyber-physical system" )
 AND
 ( "All Metadata":"pathology laboratory" OR "All Metadata":"clinical laboratory" OR "All Metadata":"core laboratory" OR "All Metadata":"medical laboratory" OR "All Metadata":"laboratory medicine" OR "All Metadata":"clinical lab" OR "All Metadata":"lab automation" ))

**3.4. Google Scholar (Horizon Scanning Stratum)**

• Search Date: May 1, 2026
• Purpose: Targeted horizon scan for emerging multi-agent systems, wet-lab agentic execution, and autonomous laboratory robotics.
• Retained Records: 10 records (tracked in Supplementary Table S1 for design derivation).

**Google Scholar Conceptual Horizon Scan:**
("Agentic workflow" OR "Multi-agent system" OR "LLM agent" OR "Autonomous agent")
AND
("laboratory automation" OR "wet lab" OR "scientific discovery" OR "self-driving laboratory")

**3.5. Manual Citation Tracking**

• Protocol: Bidirectional (backward citation snowballing from identified core pathology informatics papers, and forward citation tracking via Google Scholar / Scopus).
• Retained Records: 14 records (separately tagged as supplementary evidence).
